# Antioxidant and Anti-Protease Activities of Diazepinomicin from the Sponge-Associated *Micromonospora* Strain RV115

**DOI:** 10.3390/md10102208

**Published:** 2012-10-08

**Authors:** Usama Ramadan Abdelmohsen, Matthias Szesny, Eman Maher Othman, Tanja Schirmeister, Stephanie Grond, Helga Stopper, Ute Hentschel

**Affiliations:** 1 Julius-von-Sachs-Institute for Biological Sciences, University of Würzburg, Julius-von-Sachs-Platz 3, Würzburg 97082, Germany; Email: ute.hentschel@uni-wuerzburg.de; 2 Institute of Organic Chemistry, Eberhard-Karls-Universität, Auf der Morgenstelle 18, Tübingen 72076, Germany; Email: matthias.szesny@uni-tuebingen.de (M.S.); stephanie.grond@uni-tuebingen.de (S.G.); 3 Department of Toxicology, University of Würzburg, Würzburg 97078, Germany; Email: eman@toxi.uni-wuerzburg.de (E.M.O.); stopper@toxi.uni-wuerzburg.de (H.S.); 4 Institute of Pharmacy and Biochemistry, University of Mainz, Staudinger Weg 5, Mainz 55128, Germany; Email: schirmei@uni-mainz.de

**Keywords:** diazepinomicin, anti-protease, antioxidant, actinomycetes, *Micromonospora*

## Abstract

Diazepinomicin is a dibenzodiazepine alkaloid with an unusual structure among the known microbial metabolites discovered so far. Diazepinomicin was isolated from the marine sponge-associated strain *Micromonospora* sp. RV115 and was identified by spectroscopic analysis and by comparison to literature data. In addition to its interesting preclinical broad-spectrum antitumor potential, we report here new antioxidant and anti-protease activities for this compound. Using the ferric reducing antioxidant power (FRAP) assay, a strong antioxidant potential of diazepinomicin was demonstrated. Moreover, diazepinomicin showed a significant antioxidant and protective capacity from genomic damage induced by the reactive oxygen species hydrogen peroxide in human kidney (HK-2) and human promyelocytic (HL-60) cell lines. Additionally, diazepinomicin inhibited the proteases rhodesain and cathepsin L at an IC_50_ of 70–90 µM. It also showed antiparasitic activity against trypomastigote forms of *Trypanosoma brucei* with an IC_50 _of 13.5 µM. These results showed unprecedented antioxidant and anti-protease activities of diazepinomicin, thus further highlighting its potential as a future drug candidate.

## 1. Introduction

Diazepinomicin is a farnesylated dibenzodiazepinone that was discovered by Thallion’s DECIPHER^®^ platform [1] and was isolated for the first time from the marine *Micromonospora* strain DPJ12 cultivated from the ascidian *Didemnum proliferum* at Shishijima Island, Japan [2]. Its biosynthetic pathway was studied by McAlpine [3] and continued by Ratnayake [4]. Diazepinomicin is of considerable interest, owing to its broad-spectrum antitumor activity [5]. It showed a high inhibition potential in cancer cells *in vitro*, in tumor xenografts *in vivo*, and a high efficacy in advanced cancer patients, which was confirmed in clinical investigations. This activity is mediated by selective binding to the peripheral benzodiazepine receptor (PBR), resulting in tumor apoptosis and inhibition of the Ras/MAP kinase signaling pathway, which is involved in cellular proliferation and migration [6]. For these reasons, diazepinomicin is currently in phase II clinical trials after having successfully passed phase I clinical trials for Thallion pharmaceuticals. 

Free radicals and other reactive oxygen/nitrogen/chlorine species contribute to the development of several age-related diseases and to the aging process itself by causing oxidative stress and oxidative damage [7,8]. The implication of oxidative stress in the history of several acute and chronic clinical disorders, such as cancer, atherosclerosis and diabetes, led to the suggestion that antioxidants can be prophylactic agents against such diseases [9,10,11]. Antioxidant drugs protect from the tissue damage induced by free radicals by preventing their formation, scavenging them, or by promoting their decomposition [12]. Natural products, such as prenylated toluquinones and hydroquinones, were derived from marine sources including sponges, algae and marine microbes, and were found to exhibit good antioxidant properties [13,14,15]. 

Proteases are a big family of enzymes that are essential for growth and pathogenicity of bacteria, viruses and parasites. Therefore, they represent new potential targets for anti-infective drugs [16]. Rhodesain is expressed by the parasite *Trypanosoma brucei rhodesiense*, which is the causative agent of trypanosomiasis. Human cysteine cathepsins were reported to be associated with different mammalian tumors, suggesting their involvement in metastasis, angiogenesis and tumor progression [17]. Several protease inhibitors are in clinical phases for treatment of many diseases such as hypertension, diabetes, infectious diseases and cancer [18,19].

The goal of this study was to obtain deeper insights into the activity profile of diazepinomicin. The antioxidant activity of diazepinomicin was investigated using the chemical ferric reducing antioxidant power (FRAP) method and a cell-based assay in the human promyelocytic cell line HL-60. The cell vitality and protection from oxidative genomic damage was assessed in human kidney HK-2 cells. Moreover, the effect on the proteases rhodesain and cathepsin L was also examined by enzymatic and HPLC based assays. This work has added new potential insights to the medicinal use of diazepinomicin.

## 2. Results and Discussion

**Figure 1:**
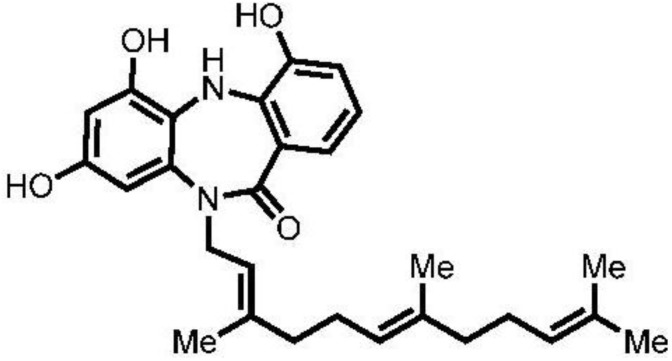
Structure of diazepinomicin (**1**).

*Micromonospora* sp. RV115 strain was isolated from the sponge *Aplysina aerophoba*, which had been collected from Rovinj, Croatia [20]. The strain was fermented in Bennett’s medium, and the secondary metabolites were collected via XAD-16 resin, which was then eluted with acetone-methanol. After dissolving the residue in water and extraction with ethyl acetate, the ethyl acetate extract was subjected to normal-phase flash chromatography with cyclohexane:ethyl acetate mixture and to final purification with reversed-phase HPLC using MeOH:H_2_O mixture supplemented with 0.05% formic acid to give compound **1** (11.1 mg). HR-ESIMS (FTICR) analysis showed that compound **1** has an [M + H]^+^ at 463.2528 and a molecular formula of C_28_H_35_N_2_O_4_. A database search using MarinLit database (2012) suggested that this mass coincided with that of diazepinomicin [2]. Analysis of ^1^H-NMR data, as well as 2D NMR data, including COSY, HSQC and HMBC spectra, and comparison with those in the literature confirmed the structure of **1** as diazepinomicin (Figure 1). 

### 2.1. Cytotoxicity of Diazepinomicin in Human Kidney Cells (HK-2)

Because reactive oxygen species (ROS) are an important mediator of renal injury [21,22], diazepinomicin (DZP) cytotoxicity was examined here in the human kidney cell line, HK-2, which was also used by others in oxidative stress studies [21]. HK-2 cells were treated with concentrations of 2–25 µM diazepinomicin for 24 h. A small and non-significant (*p* > 0.05) decrease in cell viability of less than 5% was observed, suggesting that diazepinomicin is not toxic to HK-2 cells. 

### 2.2. Antioxidant Potential of Diazepinomicin

**Figure 2:**
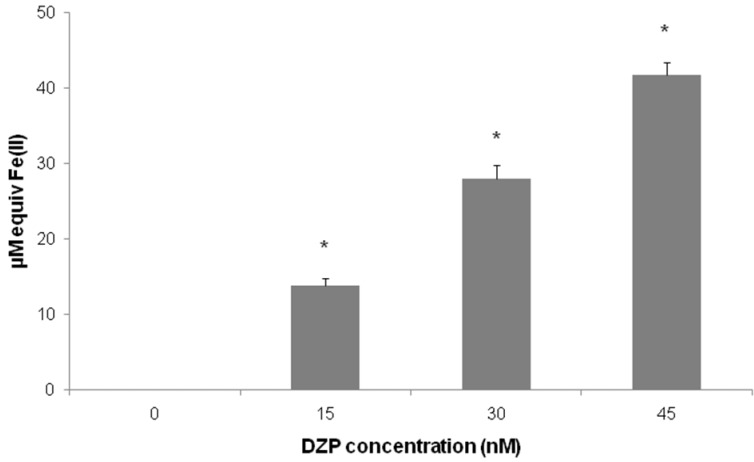
Ferric reducing antioxidant power (FRAP) of cell-free solutions of diazepinomicin assessed by the photometric quantification (*= significant *vs.* control).

The intrinsic antioxidant capacity of diazepinomicin was first assessed using the cell-free system, FRAP (Figure 2). The FRAP assay is based on the measurement of the ability of the substance to reduce Fe^3+^ to Fe^2+^, and it directly measures the reducing capacity of the compound, which is considered to be an important parameter for antioxidant function. This method reliably investigates the total antioxidant activity and has been widely used for a rapid assessment of the antioxidant potential of various food, beverages and natural products [23]. Our results demonstrate that diazepinomicin exhibited significant antioxidant activity, with 30 nM of diazepinomicin showing an equal potential to 50 µM tempol, indicating the strong antioxidant potential of diazepinomicin. 

**Figure 3:**
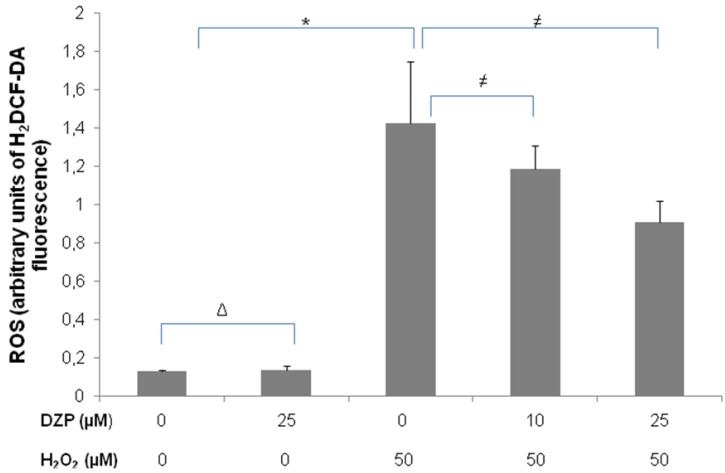
Flow cytometric analysis for the antioxidant capacity of diazepinomicin in HL-60 cells treated with 50 µM H_2_O_2_ and diazepinomicin for 30 min (Δ= non significant *vs.* control, * = significant *vs.* control and ≠ = significant *vs.* H_2_O_2_).

We further examined the antioxidant potential of diazepinomicin *in vitro* using the dye 2′,7′-dichlorodihydrofluorescein diacetate (H_2_DCF-DA). Acetate groups are removed from the molecule by cellular esterases upon entry into live cells, where the dye is oxidized to the fluorescent product dichlorofluorescein (DCF) in the presence of ROS and represents a marker for oxidative stress. The human promyelocytic cell line HL-60 was used here, because of its high sensitivity for oxidative stress. Cells were treated with diazepinomicin (10, 25 µM) in combination with 50 µM H_2_O_2 _as an oxidative stress inducer for 30 min, and the DCF fluorescence was measured using flow cytometric analysis (Figure 3). While diazepinomicin alone did not alter the ROS level in the cells, H_2_O_2_ induced an increase, which was significantly reduced by treatment with 25 µM of diazepinomicin. In this test, diazepinomicin was used at higher concentrations to compensate for the artificial oxidative stress effect induced by 50 µM H_2_O_2_. These results confirmed the assumption that diazepinomicin has high antioxidant potential. This cell-based assay, in contrast to the cell-free chemical assay FRAP (Figure 2), detects only the antioxidant compounds that can penetrate the cellular membranes of living cells and inhibit the ROS-mediated oxidation intracellularly. Marine natural products, such as aaptamine, isoaaptamine and curcudiol exhibited antioxidant potential in chemical assays but were not able to scavenge free radicals in cell-based assays [24]. This could be explained by the inability to penetrate the cell membrane, or that they are not capable of preventing 2′,7′-dichlorodihydrofluorescein oxidation intracellularly. Our results highlight the antioxidant potential of diazepinomicin by chemical and cell-based assays.

### 2.3. Protective Effect of Diazepinomicin against Oxidative Stress-Induced Cell Death in HK-2 Cells

Next, we investigated the protective effect of diazepinomicin against oxidative stress-induced cell death using H_2_O_2_. HK-2 cells were treated with 100 µM H_2_O_2_ in combination with different concentrations of diazepinomicin for 24 h (Table 1). 

**Table 1:** Cell death after 24 h incubation of HK-2 cells with 100 µM H_2_O_2,_ and different concentrations of diazepinomicin (2–25 µM) and 50 µM tempol as a positive control for antioxidant activity. (Δ= non significant increase in cell death, * = significant increase in cell death and ≠ = significant decrease in cell death).

Test substance	Cell death (% dead cells)
Control	4 ± 1.0
25 µM DZP	4.5 ± 0.0 (Δ *vs.* control )
50 µM tempol	9.7 ± 4.3
100 µM H_2_O_2_	21 ± 5.8 (* *vs.* control)
100 µM H_2_O_2_ + 2 µM DZP	6.5 ± 4.4 (≠ *vs.* H_2_O_2_)
100 µM H_2_O_2_ + 5 µM DZP	7.5 ± 1.8 (≠ *vs.* H_2_O_2_)
100 µM H_2_O_2_ + 10 µM DZP	5.3 ± 1.0 (≠ *vs.* H_2_O_2_)
100 µM H_2_O_2_ + 25 µM DZP	6.3 ± 2.5 (≠ *vs.* H_2_O_2_)
100 µM H_2_O_2_ + 50 µM tempol	14.7 ± 5.8

Our results showed that diazepinomicin (or tempol as a positive control) did not cause significant toxicity, while H_2_O_2_ led to an increased number of dead cells, which was significantly reduced in the presence of diazepinomicin. These results indicate that diazepinomicin protected HK-2 cells from H_2_O_2_-induced injury by inhibiting oxidative damage and, ultimately, cell death.

**Figure 4:**
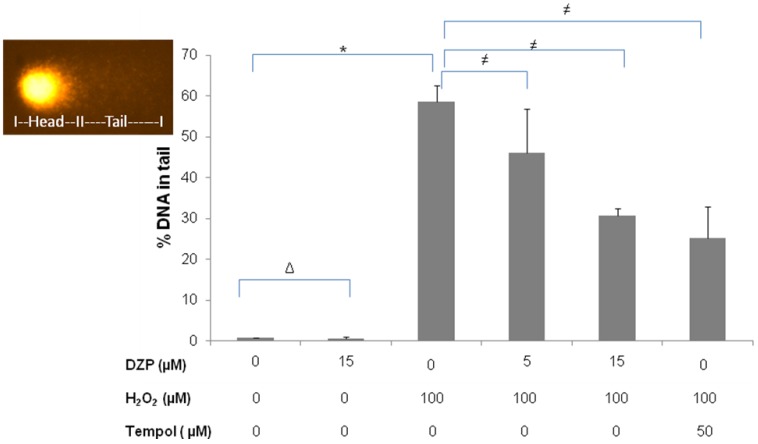
DNA damage (% DNA in tail) measured with the comet assay after treatment of HK-2 cells with 100 µM H_2_O_2,_ and diazepinomicin (5&15 µM) and 50 µM tempol for 30 min. (Δ= non significant *vs.* control, * = significant *vs.* control and ≠ = significant *vs.* H_2_O_2_).

Further investigations included the examination of the protective power of diazepinomicin against oxidative damage of DNA induced by H_2_O_2_ using the alkaline version of the comet assay (Figure 4). This endpoint detects single and double strand breaks, as well as alkali labile sites, on an individual cell basis. For this, HK-2 cells were treated with diazepinomicin or tempol in combination with 100 µM H_2_O_2_ for 30 min. While diazepinomicin alone did not cause elevated DNA damage, H_2_O_2_ yielded an increased percentage of DNA in the comet tail, which is the region containing damaged DNA. The H_2_O_2_-induced DNA damage was reduced in a dose-dependent fashion by a combination with tempol or with diazepinomicin.

### 2.4. Antitrypanosomal and Anti-Protease Activities

**Figure 5:**
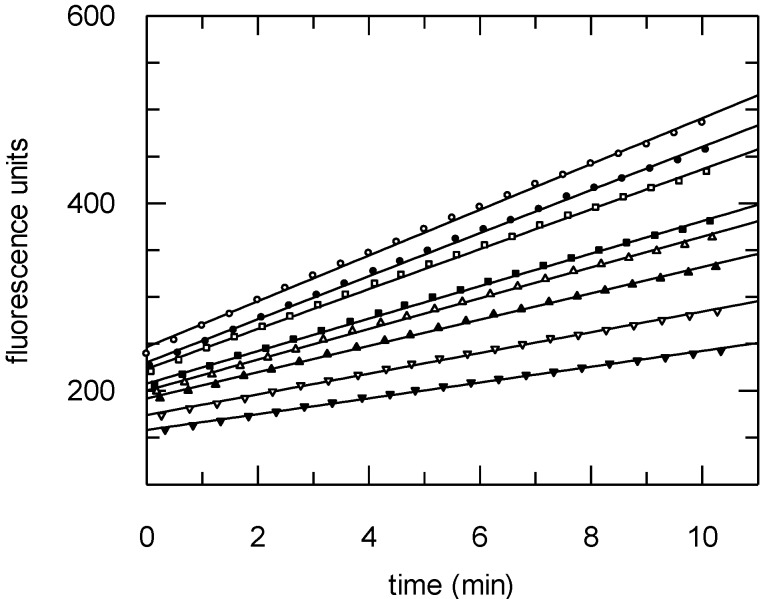
Progress curves of hydrolysis of the substrate Cbz-Phe-Arg-AMC (10 µM) in the absence or presence (from top to bottom) of diazepinomicin ([I] = 0.01 – 0.02 – 0.04 – 0.05 – 0.06 – 0.08 – 0.1 mg/mL).

**Figure 6:**
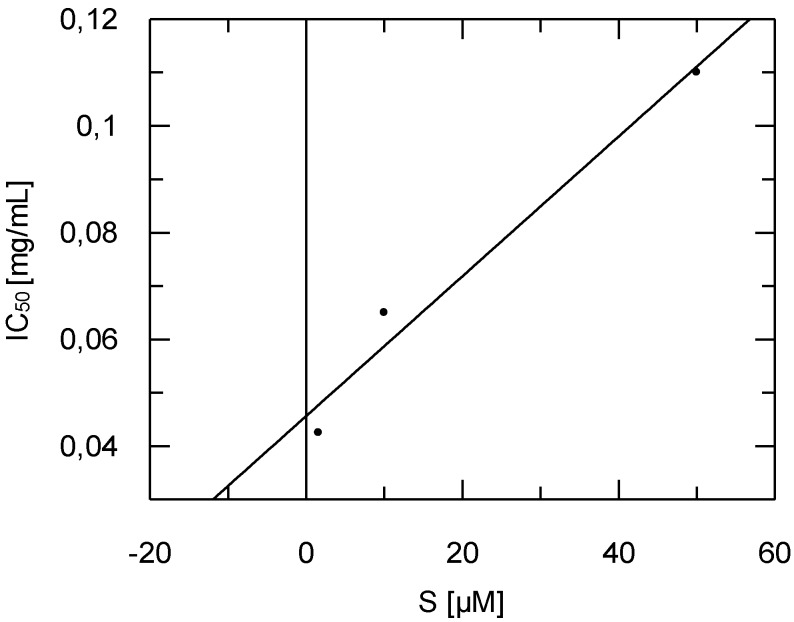
Dependency of IC_50_ values of the inhibition of rhodesain by diazepinomicin on substrate concentration. Increasing IC_50_ values at higher substrate concentrations shows diazepinomicin to be a competitive inhibitor. The *K*_i_ value (extrapolated IC_50_ at 0 substrate concentration) was determined to be 0.0456 mg/mL (98 µM).

Diazepinomicin was tested for its antitrypanosomal activity against trypomastigote forms of *Trypanosoma brucei brucei* laboratory strain TC 221 at both 48 and 72 h time points. Diazepinomicin exhibited IC_50_ values of 13.57 and 17.06 µM, respectively, which has not been previously reported in the literature to our knowledge. Since diazepinomicin exhibited activity against *Trypanosoma brucei*, we tested the compound against the parasite’s protease rhodesain. Diazepinomicin showed concentration-dependent inhibition of the hydrolysis of the fluorogenic substrate Cbz-Phe-Arg-AMC (Figure 5). Few natural products with anti-protease activities have been reported from the marine environment. One such example are the new tetromycin derivatives, tetromycins 1–4, that were isolated from *Streptomycesaxinellae* Pol001T, which had been cultivated from the Mediterranean sponge *Axinella polypoides*. Tetromycins 3–4 exhibited protease inhibition activities against several cysteine proteases [25]. Furthermore, the peptide miraziridine A that was isolated from the marine sponge *Theonella swinhoei*, exhibited potent activity against cathepsin B [26]. In the present study, the assays with the protease rhodesain at three different substrate concentrations showed diazepinomicin to be a competitive inhibitor with respect to the substrate used (Figure 6). The dissociation constant *K*_i_ of inhibition of rhodesain was determined to be 98 µM.

Since rhodesain is a parasite’s analog of the human cysteine cathepsins, which are involved in cancer progression and metastasis [27,28,29,30], we also tested the activity of diazepinomicin against human cathepsin L. Diazepinomicin showed similar activity (IC_50_ = 72.4 ± 5.3 µM at a substrate concentration of 6.25 µM from four independent assays). Since diazepinomicin quenches fluorescence of the hydrolysis product AMC (inner filter effect), correction assays were performed according to Ludewig *et al.* [31]. In order to verify the inhibition, we also detected the substrate hydrolysis by an HPLC-based method. The cathepsin L catalyzed hydrolysis of the substrate (30 min incubation time, [S] = 6.25 µM) was quantified in absence and presence of different concentrations of diazepinomicin (20, 100 µM) after column chromatographic separation of the hydrolysis product and the substrate. These studies proved the inhibitory potency of diazepinomicin (45% inhibition at 20 µM; 64% at 100 µM). We observed instability of the diazepinomicin DMSO stock solution (stored for more than 4 weeks), which was possibly due to its oxidation. Assays with solutions that were not freshly prepared showed weaker inhibitory activity and IC_50_ values of 141 µM ± 20 µM (from six independent assays) against cathepsin L. This reduced inhibitory potency with non-freshly prepared stock solutions was also seen in the HPLC-based assays with only 26% inhibition at 20 µM, and 36% inhibition at 80 µM diazepinomicin.

In conclusion, diazepinomicin exhibited antioxidant capacity using two different strategies including cell-free and cell-based assays. Diazepinomicin was able to protect cells from toxicity and genomic damage induced by the strong oxidant H_2_O_2_. This antioxidant activity will add a new perspective on the use of diazepinomicin in chemoprevention therapy for different types of cancer. Moreover, diazepinomicin showed anti-protease activity against rhodesain and cathepsin L, which suggests that anti-protease activity may contribute to the anticancer and antitrypanosomal activities as one of the possible mechanism of actions.

## 3. Experimental Section

### 3.1. Cultivation and Purification

*Micromonospora* sp. RV115 was isolated from the sponge *Aplysina aerophoba*, which was collected from the Mediterranean Sea [20]. *Micromonospora* strain RV115 was fermented in 25 × 1 L baffled flasks, each containing 200 mL of Bennett’s broth (glucose 10 g/L, beef extract 0.77 g/L, yeast extract 1 g/L, NZ-amine 2 g/L, XAD 16 resin 20 g/L in 50% ASW, pH 7.3) at 28 °C for 9 days with shaking at 180 rpm. The harvested culture broth was centrifuged (9000 rpm, 10 min, 2 °C) and the mycelium, together with the XAD 16, were extracted with acetone/methanol (3/7, 3 × 500 mL). The combined extracts were concentrated under vacuum, resuspended in deionized water (250 mL) and extracted with ethyl acetate (3 × 250 mL, pH 5). The ethyl acetate extract was purified by normal phase flash chromatography (cyclohexane, ethyl acetate starting with 100% cyclohexane, 20, 40, 60, 80 up to 100% ethyl acetate), followed by purification of the fraction with 40% ethyl acetate with reverse phase HPLC (Nucleodur 100-5 C18 ec), using a gradient 80%–100% methanol/water containing 0.05% formic acid over 20 min to yield 11.1 mg of diazepinomicin.

### 3.2. Ferric Reducing Antioxidant Power (FRAP) Assay

Total antioxidant activity was measured according to Benzie and Strain [32]. In brief, the FRAP solution was freshly prepared (25 mL of 300 mM acetate buffer, pH 3.6, 2.5 mL of 20 mM ferric chloride hexahydrate dissolved in distilled water, and 2.5 mL of 10 mM 2,4,6-tripyridyl-s-triazine (TPTZ) dissolved in 40 mM HCl). 20 μL of diazepinomicin in methanol (1 mg/mL) was mixed with 600 µL of the FRAP solution and 180 µL of water. Absorbance was measured at 595 nm after 6 min of incubation at room temperature by a spectrophotometer (Bio-Tek, Model Uvikon XL) against a blank of distilled water. Tempol was tested at the final concentration of 50 μM and used as the reference compound. FRAP values were obtained by comparing the absorbance change at 595 nm in test reaction mixtures with those containing ferrous ions in known concentrations. Absorbance changes are linear over a wide concentration range with antioxidant mixtures.

### 3.3. Cell Culture Maintenance

HK-2, a human kidney cell line with many properties of proximal tubular cells, was obtained from Dr. G. Garibotto, Department of Internal Medicine and Urology, University of Genoa, Italy. Cells were cultured two times per week at 37 °C in DMEM/F12 medium (PAA Laboratories GmbH, Pasching, Austria) supplemented with 5% fetal calf serum, 2 mM of L-glutamine, 1% antibiotics (50 U/mL penicillin, 50 mg/mL streptomycin), 10 µg/L epidermal growth factor, 5 µg/L hydrocortisone, 5 µg/L sodium selenate, 5 ng/L bovine pituitary extract, 5 mg/L transferrin, and 5 mg/L insulin and 5 ng/L T3. 

HL-60, a human promyelocytic cell line was donated by Prof. R. Schinzel, Vasopharm GmbH, Würzburg, Germany. HL-60 cells were cultured three times per week at 37 °C, 5% (v/v) CO_2_ in RPMI 1640 medium, supplemented with 10% (v/v) fetal bovine serum (FBS), 1% (w/v) L-glutamine and 0.4% (w/v) antibiotics (50 U/mL penicillin, and 50 mg/mL streptomycin). 

### 3.4. Flow Cytometric Quantification of Antioxidant Activity

The potential of antioxidant activity was estimated using the probe 2′,7′-dichlorodihydrofluorescein diacetate (H_2_DCF-DA). The cell-permeable H_2_DCF-DA is non-fluorescent until the acetate groups are removed by intracellular esterases and until oxidation occurs within the cell, leading to the fluorescent product dichlorofluorescein (DCF). Oxidation of this probe can be detected by monitoring the increase in fluorescence using flow cytometer with filters appropriate for fluorescein (FITC). HL-60 cells (5 × 10^5^) were seeded in 25 cm^2^ flasks and incubated in medium containing 50 µM H_2_O_2_, 25 µM diazepinomicin or H_2_O_2_ with diazepinomicin (10 and 25 µM) for 30 min. At the last 10 min of the treatment, 10 μM H_2_DCF-DA were additionally loaded. Afterwards, the medium was removed, the cells were rinsed with PBS, and 1 mL of BSA/PBS was added per flask. Fluorescence was measured at λ_exc_ 475 nm; λ_em_ 525 nm. To determine the viability of the cells during the process, samples were subsequently incubated with 0.1% (v/v) 50 μM PI, incubated for 10 min at room temperature, and the fluorescence (λ_exc_ 538 nm, λ_em_ 590 nm) was measured. Results are expressed as the ratio DCF (dichlorofluorescein) fluorescence/PI fluorescence.

### 3.5. Vitality Test

The assay was performed as described by Schmitt *et al.* [33]. However, ethidium bromide was replaced with Gel RedBiotrend (Köln, Germany). Vitality staining was performed for the HK-2 cells treated with different concentrations of diazepinomicin (2 to 25 µM) for 24 h. 0.35 × 10^6^ cells were seeded in 25 cm^2^ flasks for 24 h in a control medium. After treatment with diazepinomicin or tempol as positive control, cells were harvested, and 70 μL of the cell suspension was stained with 30 μL staining solution. Twenty microliter of this mixture was applied to the slide, and the fractions of green and red cells in a total of 200 cells were counted at a 500-fold magnification with a fluorescence microscope.

### 3.6. Comet Assay

After treatment of HK-2 cells with 100 µM H_2_O_2_ alone, or with H_2_O_2_ and diazepinomicin (5 and 15 µM), the cells were harvested, and 20 μL of the treated cell suspension were mixed with 180 μL of 0.5% low melting agarose and added to fully frosted slides that had been covered with a bottom layer of 1% normal melting point agarose. The slides were incubated in lysis solution (2.5 M NaCl, 0.1 M EDTA, 0.01 M Tris and 1% Triton X-100, 10 g/L *N*-lauroylsarcosine sodium adjusted to pH 10 with NaOH) at 4 °C. After 1 h, the slides were washed and then placed in the electrophoresis solution (300 mM NaOH, 1 mM EDTA, pH > 13.0) for 20 min. The electrophoresis was conducted for 20 min at 25 V (1.1 V/cm) and 300 mA. The slides were neutralized in 0.4 M Tris buffer (pH 7.5) and then dehydrated in methanol for 10 min at −20 °C. The slides were left at 37 °C in an incubator to dry and then stored at room temperature. Before evaluation, 20 μL of Gel red /DABCO solution was added to each slide. Images of 50 randomly selected cells (25 per replicate slide) for each sample were analyzed with a fluorescence microscope (Labophot 2, Nikon, Germany) at 200-fold magnification using image analysis software (Komet 5, BFI Optilas, Germany). A representative picture of a damaged cell in the comet assay is inserted into Figure 4. While intact nuclear DNA remains in the head region of the cellular comet, damaged DNA moves faster during electrophoresis and, thus, forms the tail region of the cellular comet. The percentage of DNA in the tail region was used to quantify DNA damage-related migration.

### 3.7. Statistics for Cell Based Assays

Data from 3 independent experiments ± standard deviation are depicted. Statistical significance among multiple groups was tested with the Kruskal-Wallis test. Individual groups were then tested using the Mann Whitney U-test, and results were considered significant if the *p*-value was ≤ 0.05.

### 3.8. Antitrypanosomal Activity

Antitrypanosomal assay was done as described by Huber and Koella [34]. In brief, trypomastigote forms of *Trypanosoma brucei brucei* laboratory strain TC 221 were cultured in Complete Baltz medium [35], and a defined number of parasites (10^4^ trypanosomes per mL) were exposed in test chambers of 96-well plates to various concentrations of diazepinomicin (previously dissolved in DMSO) to make a final volume of 200 μL in duplicates. Trypanosomes in a culture medium as a positive control, and diazepinomicin without trypanosomes as a negative control were run simultaneously with each plate. After incubating the plates at 37 °C in an atmosphere of 5% CO_2_ for a total time period of 72 h, 20 μL of Alamar Blue was added and the activity was measured by light absorption using Microplate Reader MR 700 at a wavelength of 550 nm with a reference wave length of 630 nm. First reading was done at 48 h and subsequently at 72 h. The effect of the test substances was quantified in IC_50_ values by linear interpolation of three independent measurements.

### 3.9. Protease Assays

The assays were performed as previously described [36]. In brief, the hydrolysis of Cbz-Phe-Arg-AMC by either rhodesain or human cathepsin L was detected continuously over a period of 10 min using a Varian Cary Eclipse spectrofluorometer (Varian, Darmstadt, Germany) with a microplate reader (excitation at 365 nm, emission at 460 nm, 25 °C). The following buffer solutions were used: 50 mM sodium acetate buffer, pH 5.5 containing 2 mM (cathepsin L), or 5 mM (rhodesain) DTT, 5 mM EDTA, 200 mM NaCl and 0.005% Brij 35. DMSO stock solutions of substrate and inhibitor were freshly prepared. DMSO alone (7.5% final concentration) was used as a negative control.

For the HPLC based assay, the same conditions were used: the enzymatic hydrolysis of the substrate in absence or presence of diazepinomicin was stopped after 30 min by diluting the reaction with acetonitrile. The reaction mixtures (50 µL) were submitted to HPLC, and the amounts of AMC produced and of residual substrate Cbz-Phe-Arg-AMC were detected after separation using a Waters symmetry C18 column (3.5 µm, 4.6 × 75 mm) and a gradient with the solvents, A: phosphate buffer 25 mM pH 6.0 with 10% methanol; and B: methanol (from 10% A/90% B to 100% A/0% B within 40 min, flow 0.8 mL/min). A diode array detector (DAD) was used (210–400 nm). The AMC peak (retention time 2 min) was quantified (area under the curve, AUC) at 347 nm, whereas the substrate peak (retention time 30 min) was quantified at 326 nm. The percentage of inhibition by diazepinomicin was calculated for each experiment comparing the AUCs of the substrate peaks without enzyme, after 30 min incubation of substrate and enzyme, after 30 min incubation of substrate, enzyme and inhibitor, and by comparing the AUCs of the AMC peaks after 30 min incubation of substrate and enzyme with those obtained after 30 min incubation of substrate, enzyme and inhibitor. Each experiment was performed in triplicate.

## 4. Conclusions

Our results showed that diazepinomicin exhibited antioxidant capacity using two different strategies including cell-free and cell-based assays. Diazepinomicin was able to protect cells from toxicity and genomic damage induced by the strong oxidant H_2_O_2_. This antioxidant activity will add a new perspective on the use of diazepinomicin in chemoprevention therapy for different types of cancer. Moreover, diazepinomicin showed anti-protease activity against rhodesain and cathepsin L, which suggests that anti-protease activity may contribute to the anticancer and antitrypanosomal activities as one of the possible mechanism of actions.

## References

[1] Zazopoulos E., Huang K., Staffa A., Liu W., Bachmann B.O., Nonaka K., Ahlert J., Thorson J.S., Shen B., Farnet C.M. (2003). A genomics-guided approach for discovering and expressing cryptic metabolic pathways. Nat. Biotechnol..

[2] Charan R.D., Schlingmann G., Janso J., Bernan V., Feng X., Carter G.T. (2004). Diazepinomicin, a new antimicrobial alkaloid from a marine *Micromonospora* sp. J. Nat. Prod..

[3] McAlpine J.B., Banskota A.H., Charan R.D., Schlingmann G., Zazopoulos E., Piraee M., Janso J., Bernan V.S., Aouidate M., Farnet C.M. (2008). Biosynthesis of diazepinomicin/ECO-4601, a *Micromonospora* secondary metabolite with a novel ring system. J. Nat. Prod..

[4] Ratnayake A.S., Janso J.E., Feng X., Schlingmann G., Goljer I., Carter G.T. (2009). Evaluating indole-related derivatives as precursors in the directed biosynthesis of diazepinomicin analogues. J. Nat. Prod..

[5] Campas C. (2009). Diazepinomicin. Drug Fut..

[6] Wong K.K. (2009). Recent developments in anti-cancer agents targeting the Ras/Raf/ MEK/ERK pathway. Recent Pat. Anticancer Drug Discov..

[7] Halliwell B., Gutteridge J. (1999). Free Radicals in Biology and Medicine.

[8] Sohal R.S., Mockett R.J., Orr W.C. (2002). Mechanisms of aging: an appraisal of the oxidative stress hypothesis. Free Radic. Biol. Med..

[9] Chowienczyk P.J., Brett S.E., Gopaul N.K., Meeking D., Marchetti M., Russell-Jones D.L., Anggard E.E., Ritter J.M. (2000). Oral treatment with an antioxidant (raxofelast) reduces oxidative stress and improves endothelial function in men with type II diabetes. Diabetologia.

[10] Parthasarathy S., Santanam N., Ramachandran S., Meilhac O. (2000). Potential role of oxidized lipids and lipoproteins in antioxidant defense. Free Radic. Res..

[11] DeNicola G.M., Karreth F.A., Humpton T.J., Gopinathan A., Wei C., Frese K., Mangal D., Yu K.H., Yeo C.J., Calhoun E.S. (2011). Oncogene-induced Nrf2 transcription promotes ROS detoxification and tumorigenesis. Nature.

[12] Young I.S., Woodside J.V. (2001). Antioxidants in health and disease. J. Clin. Pathol..

[13] Zhang C.Y., Wu W.H., Wang J., Lan M.B. (2012). Antioxidant properties of polysaccharide from the brown seaweed *Sargassum graminifolium* (Turn.), and its effects on calcium oxalate crystallization. Mar. Drugs.

[14] Song L., Li T., Yu R., Yan C., Ren S., Zhao Y. (2008). Antioxidant activities of hydrolysates of Arca subcrenata prepared with three proteases. Mar. Drugs.

[15] Sunassee S.N., Davies-Coleman M.T. (2012). Cytotoxic and antioxidant marine prenylated quinones and hydroquinones. Nat. Prod. Rep..

[16] Zhang C., Kim S.K. (2009). Matrix metalloproteinase inhibitors (MMPIs) from marine natural products: The current situation and future prospects. Mar. Drugs.

[17] Hsieh C.C., Hernandez-Ledesma B., Jeong H.J., Park J.H., de Lumen B.O. (2010). Complementary roles in cancer prevention: Protease inhibitor makes the cancer preventive peptide lunasin bioavailable. PLoS One.

[18] Cai H., Kuang R., Gu J., Wang Y. (2011). Proteases in malaria parasites—a phylogenomic perspective. Curr. Genomics.

[19] McKerrow J.H., Rosenthal P.J., Swenerton R., Doyle P. (2008). Development of protease inhibitors for protozoan infections. Curr. Opin. Infect. Dis..

[20] Abdelmohsen U.R., Pimentel-Elardo S.M., Hanora A., Radwan M., Abou-El-Ela S.H., Ahmed S., Hentschel U. (2010). Isolation, phylogenetic analysis and anti-infective activity screening of marine sponge-associated actinomycetes. Mar. Drugs.

[21] Verzola D., Bertolotto M.B., Villaggio B., Ottonello L., Dallegri F., Salvatore F., Berruti V., Gandolfo M.T., Garibotto G., Deferrari G. (2004). Oxidative stress mediates apoptotic changes induced by hyperglycemia in human tubular kidney cells. J. Am. Soc. Nephrol..

[22] Djamali A. (2007). Oxidative stress as a common pathway to chronic tubulointerstitial injury in kidney allografts. Am. J. Physiol. Renal Physiol..

[23] Moyer R.A., Hummer K.E., Finn C.E., Frei B., Wrolstad R.E. (2002). Anthocyanins, phenolics, and antioxidant capacity in diverse small fruits: Vaccinium, rubus, and ribes. J. Agric. Food Chem..

[24] Takamatsu S., Hodges T.W., Rajbhandari I., Gerwick W.H., Hamann M.T., Nagle D.G. (2003). Marine natural products as novel antioxidant prototypes. J. Nat. Prod..

[25] Pimentel-Elardo S.M., Buback V., Gulder T.A., Bugni T.S., Reppart J., Bringmann G., Ireland C.M., Schirmeister T., Hentschel U. (2011). New tetromycin derivatives with anti-trypanosomal and protease inhibitory activities. Mar. Drugs.

[26] Tabares P., Degel B., Schaschke N., Hentschel U., Schirmeister T. (2012). Identification of the protease inhibitor miraziridine A in the Red sea sponge *Theonella swinhoei*. Pharmacogn. Res..

[27] Leto G., Sepporta M.V., Crescimanno M., Flandina C., Tumminello F.M. (2010). Cathepsin L in metastatic bone disease: therapeutic implications. Biol. Chem..

[28] Yan J.A., Xiao H., Ji H.X., Shen W.H., Zhou Z.S., Song B., Chen Z.W., Li W.B. (2010). Cathepsin L is associated with proliferation and clinical outcome of urothelial carcinoma of the bladder. J. Int. Med. Res..

[29] Colella R., Lu G., Glazewski L., Korant B., Matlapudi A., England M.R., Craft C., Frantz C.N., Mason R.W. (2010). Induction of cell death in neuroblastoma by inhibition of cathepsins B and L. Cancer Lett..

[30] Joyce J.A., Baruch A., Chehade K., Meyer-Morse N., Giraudo E., Tsai F.Y., Greenbaum D.C., Hager J.H., Bogyo M., Hanahan D. (2004). Cathepsin cysteine proteases are effectors of invasive growth and angiogenesis during multistage tumorigenesis. Cancer Cell.

[31] Ludewig S., Kossner M., Schiller M., Baumann K., Schirmeister T. (2010). Enzyme kinetics and hit validation in fluorimetric protease assays. Curr. Top. Med. Chem..

[32] Benzie I.F., Strain J.J. (1999). Ferric reducing/antioxidant power assay: direct measure of total antioxidant activity of biological fluids and modified version for simultaneous measurement of total antioxidant power and ascorbic acid concentration. Methods Enzymol..

[33] Schmitt E., Lehmann L., Metzler M., Stopper H. (2002). Hormonal and genotoxic activity of resveratrol. Toxicol. Lett..

[34] Huber W., Koella J.C. (1993). A comparison of three methods of estimating EC_50_ in studies of drug resistance of malaria parasites. Acta Trop..

[35] Baltz T., Baltz D., Giroud C., Crockett J. (1985). Cultivation in a semi-defined medium of animal infective forms of *Trypanosoma brucei*, *T*. *equiperdum*, *T*. *evansi*, *T*. *rhodesiense* and *T*. *gambiense*. EMBO J..

[36] Breuning A., Degel B., Schulz F., Buchold C., Stempka M., Machon U., Heppner S., Gelhaus C., Leippe M., Leyh M. (2010). Michael acceptor based antiplasmodial and antitrypanosomal cysteine protease inhibitors with unusual amino acids. J. Med. Chem..

